# Ultradian Secretion of Growth Hormone in Mice: Linking Physiology With Changes in Synapse Parameters Using Super-Resolution Microscopy

**DOI:** 10.3389/fncir.2020.00021

**Published:** 2020-05-25

**Authors:** Klaudia Bednarz, Walaa Alshafie, Sarah Aufmkolk, Théotime Desserteaux, Pratap Singh Markam, Kai-Florian Storch, Thomas Stroh

**Affiliations:** ^1^Department of Neurology and Neurosurgery, Montreal Neurological Institute, McGill University, Montreal, QC, Canada; ^2^Department of Anatomy and Cell Biology, McGill University, Montreal, QC, Canada; ^3^Integrated Program in Neuroscience, McGill University, Montreal, QC, Canada; ^4^Department of Genetics, Harvard Medical School, Boston, MA, United States; ^5^Douglas Mental Health University Institute, Montreal, QC, Canada; ^6^Department of Psychiatry, McGill University, Montreal, QC, Canada

**Keywords:** *d*STORM, SMLM, growth hormone, structural synaptic study, neuroendocrine circuits

## Abstract

Neuroendocrine circuits are orchestrated by the pituitary gland in response to hypothalamic hormone-releasing and inhibiting factors to generate an ultradian and/or circadian rhythm of hormone secretion. However, mechanisms that govern this rhythmicity are not fully understood. It has been shown that synaptic transmission in the rodent hypothalamus undergoes cyclical changes in parallel with rhythmic hormone secretion and a growing body of evidence suggests that rapid rewiring of hypothalamic neurons may be the source of these changes. For decades, structural synaptic studies have been utilizing electron microscopy, which provides the resolution suitable for visualizing synapses. However, the small field of view, limited specificity and manual analysis susceptible to bias fuel the search for a more quantitative approach. Here, we apply the fluorescence super-resolution microscopy approach *direct* Stochastic Optical Reconstruction Microscopy (*d*STORM) to quantify and structurally characterize excitatory and inhibitory synapses that contact growth hormone-releasing-hormone (GHRH) neurons during peak and trough values of growth hormone (GH) concentration in mice. This approach relies on a three-color immunofluorescence staining of GHRH and pre- and post-synaptic markers, and a quantitative analysis with a Density-Based Spatial Clustering of Applications with Noise (DBSCAN) algorithm. With this method we confirm our previous findings, using electron microscopy, of increased excitatory synaptic input to GHRH neurons during peak levels of GH. Additionally, we find a shift in synapse numbers during low GH levels, where more inhibitory synaptic inputs are detected. Lastly, we utilize *d*STORM to study novel aspects of synaptic structure. We show that more excitatory (but not inhibitory) pre-synaptic clusters associate with excitatory post-synaptic clusters during peaks of GH secretion and that the numbers of post-synaptic clusters increase during high hormone levels. The results presented here provide an opportunity to highlight *d*STORM as a valuable quantitative approach to study synaptic structure in the neuroendocrine circuit. Importantly, our analysis of GH circuitry sheds light on the potential mechanism that drives ultradian changes in synaptic transmission and possibly aids in GH pulse generation in mice.

## Introduction

Growth Hormone (GH) is a major regulator of longitudinal growth during childhood and puberty, and of anabolic metabolism in mammals throughout life. It is released in a pulsatile fashion with major episodes of GH secretion occurring at approximately 3-h intervals followed by prolonged trough periods of near undetectable basal serum GH levels in most species (Tannenbaum and Martin, 1976; Bertherat et al., 1995). This rhythm of GH secretion appears to be generated by the interaction of two hypothalamic neuropeptides, excitatory GH-Releasing Hormone (GHRH) and inhibitory somatostatin (SOM, a.k.a. Somatotropin Release-Inhibiting Factor, SRIF) in the hypophysis and in the hypothalamic arcuate nucleus (ARC). In male rodents, these two regulatory peptides are released in reciprocal 3–4 h cycles in the median eminence of the hypothalamus, where they enter the hypophyseal portal blood supply and thereby reach somatotropes in the anterior lobe of the hypophysis (Tannenbaum and Ling, 1984; Plotsky and Vale, 1985). It has been hypothesized that SOM is released into the ARC with the same periodicity (Wagner et al., 1998). Recently, we have also shown that the release of the stimulating hormone from the hypothalamus together with the cellular localization of SOM receptors in pituitary cells fine-tunes the rhythms of pituitary hormone secretion (Alshafie et al., 2019).

In parallel with this ultradian cycle of GH secretion and reciprocal secretion of regulatory hypothalamic neuropeptides, it was found that the binding of radiolabeled SOM to the ARC in rodents also oscillates, exhibiting the same periodicity as GH secretion (Tannenbaum et al., 1993). This led to the hypothesis that differential plasma membrane targeting of somatostatin receptors, which are increasing the susceptibility of GHRH neurons in the ARC to inhibition by locally released SOM, may be underlying the observed ultradian rhythm of GH secretion at the hypothalamic level (Tannenbaum et al., 1993; Stroh et al., 2009). However, later electron microscopic studies in fixed tissue from rats with known plasma GH status on the subcellular localization of SOM receptors in GHRH neurons of the ARC revealed that there was no significant difference in the ratio of intracellular vs. plasma membrane-bound receptors in a GH trough vs. peak (Stroh et al., 2009). Interestingly, the relative abundance of excitatory vs. inhibitory synapses, as identified by their symmetric, i.e., inhibitory, vs. asymmetric, i.e., excitatory, morphology (Peters et al., 1991), contacting dendritic profiles of GHRH neurons, varied markedly between the phases of the GH secretion cycle, with a predominance of inhibitory synapses during trough periods. This predominance was not found during GH peak periods. However, the mechanisms behind that observation remained unknown and the small numbers dictated by the technique used, transmission electron microscopy (TEM), made the quantification and interpretation of the finding difficult.

New fluorescence microscopy methods that bisect the diffraction limit of optical resolution have summarily been termed super-resolution microscopy (Sigal et al., 2018). Single molecule localization microscopy (SMLM) in particular, permits nanoscopic resolution by clever exploitation of the photo-chemical properties of fluorophores. Identifying proteins of interest with a resolution down to 10 nm makes SMLM a competitive tool compared to TEM (Baddeley and Bewersdorf, 2018). Here, we use the SMLM technique of *direct* Stochastic Optical Reconstruction Microscopy (*d*STORM), by labeling proteins of interest with commercially available antibodies. The samples are immersed in a chemical environment favoring reversible depletion of fluorescent events enabling single fluorophore detection (Bates et al., 2007; Heilemann et al., 2008; Baddeley and Bewersdorf, 2018). This method is well established in the neuroimaging field and has been used to reveal unknown characteristics of the neuronal ultrastructure. For instance, it was shown that axons exhibit a para-crystalline arrangement of the actin cytoskeleton in which actin forms regularly spaced rings around the circumference of the axon at approximately 180 nm periodicity. Spectrin forms alternating structures with the actin rings (Xu et al., 2013; Lorenzo et al., 2019). Studies like (Nair et al., 2013; Andreska et al., 2014; Ehmann et al., 2014; Rahbek-Clemmensen et al., 2017; Siddig et al., 2020) emphasized the quantitative potential of dual-color *d*STORM by quantifying synaptic protein distribution in synapses in primary cell culture, *Drosophila* larvae and murine cerebellum tissue samples.

In the present study, we hypothesized that the change in the relative abundance of excitatory vs. inhibitory synapses contacting GHRH neurons in the ARC in parallel with the GH secretion cycle (Stroh et al., 2009) may be a result of physical uncoupling of excitatory presynaptic terminals from their postsynaptic counterparts. To test this hypothesis, we used mice from which plasma GH levels had been measured continuously at high temporal resolution using a highly sensitive sandwich ELISA (Steyn et al., 2011) to determine which animals were in either a GH secretory episode or trough at the time of fixation. Brain sections from these two groups were then stained for pre- and post-synaptic markers of excitatory or inhibitory central synapses, and a GHRH marker to identify synapses on GHRH-positive neurons. These samples were then imaged using *d*STORM, which enabled the measurement of multiple morphological parameters at the ultrastructural level and detailed quantification of the data using cluster analysis algorithms.

## Materials and Methods

### Measures of Pulsatile GH Secretion and Tissue Extraction

**Figure 1:**
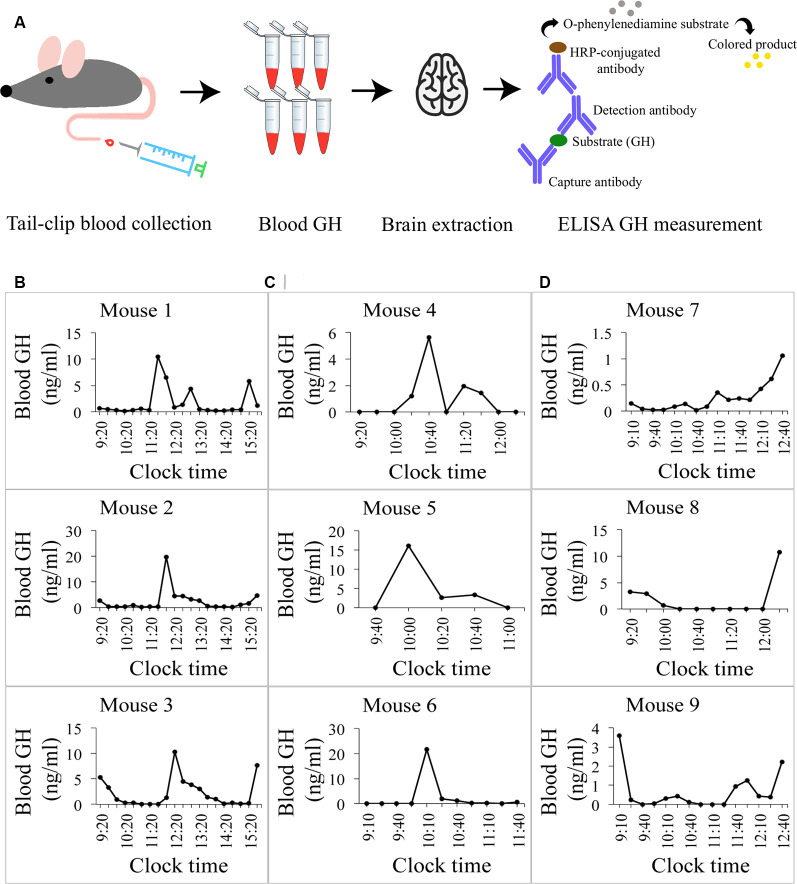
Growth hormone (GH) profiles in mice. **(A)** A pictogram of the data collection for blood GH levels. Blood was collected from the mouse’s tail at regular intervals to obtain profiles of circulating GH concentrations. The mouse was then perfused and the brain extracted. A sandwich-ELISA GH measurement from the collected blood samples followed. **(B)** Daily profiles of circulating GH levels in ng/ml in three different mice. Oscillating levels of GH are observed with a peak of GH secretion around the same time (~13:20) and baseline levels of GH at 0 ng/ml in all mice. Detection minimum from the standard curve = 0.2 ng/ml, ELISA essay detection sensitivity 0.03 ng/ml. **(C)** Circulating GH levels in mice sacrificed at the time of baseline hormone secretion (0 ng/ml). The last point in each graph represents the final blood collection point. **(D)** Circulating GH levels in mice sacrificed at the time of high hormone secretion. Final blood GH concentrations range from 1.04 ng/ml to 11 ng/ml.

All animal procedures were approved by the Animal Care Committee of McGill University and conducted in compliance with the guidelines of the Canadian Council of Animal Care. Pulsatile GH secretion measurements were done as described by Steyn et al. (2011). Eighteen adult male (8–10 weeks-old) C57BL/6 mice (Charles River Canada, Saint Constant, QC, Canada) were group-housed (*n* = 4) for at least 2 weeks before the experiment under a 12 h light, 12 h dark cycle (lights on at 08:00 a.m. and off at 08:00 p.m.). Room temperature was maintained at 20 ± 2°C. Mouse chow and tap water were available *ad libitum*. To minimize stress, prior to all experiments, mice were habituated to the test environment (human handling and tail manipulation) for 10 days, 15 min each day. Habituation was done by the same person conducting all subsequent experiments. A small cardboard tube was placed in the cage 2 days before the sample collection and used during experimental procedures to assist in animal handling. Starting at 09:10, 09:20 or 09:40 a.m., sequential tail-clip blood collections were performed in 15 or 20-min intervals from each mouse, where mice were placed inside the cardboard tube and ~2 mm of the distal portion of the tail was excised once, at the beginning of the experiment, using a surgical blade. A small volume of whole blood (2 μl) was collected with a pipette and transferred to an Eppendorf tube containing 58 μl of 0.05% phosphate-buffered saline (PBS)-Tween 20 and placed on dry ice until the end of the collection period. Gentle pressure was applied with a gauze to the wound to stop the blood flow and the mouse was placed back in its cage. For subsequent blood withdrawals, the surface of the original wound was disrupted (mechanically or by applying gauze soaked in physiological saline). Collection of each sample was performed in less than 45 s. After collecting several blood samples from each mouse (5–15 samples, end times chosen randomly), the mouse was anesthetized with an intraperitoneal injection of an anesthetic cocktail (Ketamine/Xylazine/Acepromazine), followed by a trans-aortic perfusion with 4% paraformaldehyde (PFA) in 0.1 M sodium-phosphate buffer (SPB), consisting of Na_2_HPO_4_ and NaH_2_PO_4_ in ddH_2_O at pH 7.4. The time delay between the last blood collection and brain extraction was 15 min. Brains were removed immediately and post-fixed in the same fixative overnight at 4°C. The next day, brains were placed in 30% sucrose solution in 0.1 M SPB overnight, snap-frozen at −40°C in isopentane and stored at −80°C until sectioning. 30 μm coronal sections were obtained with a Leica Ultramicrotome and stored in an antifreeze solution (30% glycerol + 30% ethylene glycol in 0.12 M PBS) until *d*STORM imaging. Plasma growth hormone (GH) concentrations were measured using a modified version of the sensitive sandwich ELISA assay, originally described in Steyn et al. (2011). As such, a 96-well plate (Corning Inc., 9018) was coated overnight at 4°C with 50 μl monkey anti-rat GH antibody (AFP411S, NIDDK-NHPP, Torrance, CA, USA) at a final dilution of 1:40,000. Each well was subsequently incubated with 200 μl blocking buffer (5% skim milk powder in 0.05% PBS-Tween 20) for 2 h at room temperature. A standard curve was generated using 2-fold serial dilutions of mouse GH (reference preparation, AFP-10783B, NIDDK-NHPP) in 0.05% PBS-Tween 20 supplemented with 1 ng/ml normal goat serum (NGS) to a final concentration of 0.2% NGS-Tween 20. Fifty microliter of standard curve solutions in duplicates or blood samples in singlets were loaded to the plate and incubated for 2 h at room temperature on an orbital shaker. After washing, bound standards and samples were incubated with 50 μl detection antibody (rabbit antiserum to rGH, AFP5672099, NIDDK-NHPP) at a final dilution of 1:40,000 in a blocking buffer for 90 min. The bound complex was incubated with 50 μl horseradish peroxidase-conjugated antibody (goat anti-rabbit, BioRad, Berkley, CA, USA) at a final dilution of 1:2,000 in blocking buffer for 90 min. Addition of 100 μl O-phenylenediamine (00-2003, Invitrogen, Carlsbad, CA, USA) substrate to each well resulted in an enzymatic colorimetric reaction. This reaction was stopped by addition of 50 μl 3 M HCl, and the absorbance was read at dual wavelengths of 490 nm and 650 nm with a microplate reader. The concentration of GH in each well was calculated by the regression of the standard curve using Graph Pad Prism. Figure 1A graphically represents the blood collection, brain extraction and ELISA procedures.

### Immunohistochemistry

Thirty micrometer mouse brain sections representing approximately Bregma –1.06 mm to –2.54 mm of a mouse brain atlas by Franklin and Paxinos (2012), containing the tuberal hypothalamus with the arcuate nucleus (harboring GHRH neurons) were used for all experiments. The sections were selected visually, according to their position within the brain and tissue morphology (shape of the third ventricle, position of lateral ventricles, distinct mound of the median eminence). Prior to immunofluorescence staining, each section was washed with 0.1 M SPB for 3 h to remove the antifreeze solution and incubated with 0.1% NaBH_4_ in 0.1 M tris-buffered saline (TBS) to quench any autofluorescence from residual PFA. The sections were then washed three times in 0.1 M TBS and incubated in 500 μl blocking buffer containing 10% NGS, 3% BSA and 0.1% Triton X-100 in 0.1 M TBS for 2 h. This was followed by incubation with 300 μl primary antibody solution containing primary antibodies, 0.1% Triton X-100 and 2% NGS in 0.1 M TBS at 4°C overnight. Each section was labelled addressing three targets including a pair of antibodies against a pre- and post-synaptic marker of either inhibitory or excitatory synapses plus an antibody against Growth-Hormone Releasing Hormone (GHRH; Table 1). Control sections were incubated with two of the three primary antibodies. The following day, sections were washed three times for 5 min with 0.1 M TBS + 0.1% Triton X-100, then incubated with secondary antibody solution containing appropriate species-specific secondary antibodies (Table 1), 0.1% Triton X-100 and 2% NGS in 0.1 M TBS for 1.5 h. Control sections were incubated in the same manner with all three secondary antibodies. Next, sections were washed twice in a solution of 0.1 M TBS and 0.1% Triton X-100 for 5 min and three more times for 5 min with 0.1 M TBS. Sections were then post-fixed in 4% PFA in 0.1 M SPB for 30 min to immobilize the antibodies and thus avoid movement during imaging, then washed three times for 10 min in 0.1 M SPB. Finally, to reduce light scattering and allow for 3D tissue imaging, sections were stored in Scale U2 tissue clearing buffer (30% v/v glycerol, 4 M urea, 0.1% Triton X-100; Hama et al., 2011), minimum overnight at 4°C and until *d*STORM imaging (for a maximum of 7 days).

**Table 1:** List of antibodies used in the study.

Primary antibodies						
**Antigen**	**Target details**	**Clone**	**Host**	**Supplier**	**Product code**	**Dilution**
GHRH	GHRH hormone in soma	Polyclonal	Rabbit	AbClonal	A5343	1:200
VGAT	Inhibitory pre-synaptic terminal	Polyclonal	Guinea Pig	Synaptic Systems	131004	1:1000
Gephyrin	Inhibitory post-synaptic terminal	Monoclonal	Mouse	Synaptic Systems	147021	1:500
VGLUT2	Excitatory pre-synaptic terminal	Polyclonal	Guinea Pig	Synaptic Systems	135403	1:5000
PSD-95	Excitatory post-synapftic terminal	Polyclonal	Mouse	Abcam	ab18258	1:500
**Secondary antibodies**
**Antigen**	**Conjugation**	**Host**	**Supplier**	**Product code**	**Dilution**	
Rabbit IgG	Alexa Fluor 488	Goat	Jackson ImmunoResearch	111545003	1:500	
Mouse IgG	CF 568	Goat	Biotum	20100	1:800	
Guinea Pig IgG	Alexa Fluor 647	Goat	Life Technologies	A21236	1:1000	

### Optical Set-Up for *d*STORM Imaging and 3D Multi-color Calibration

All super-resolution imaging was performed with the commercial microscope Vutara 350 (Bruker Corp., Billerica, MA, USA), equipped with Single Molecule Localization (SML) biplane technology (Juette et al., 2008). The microscope is equipped with a Hamamatsu high resolution (4 MP, 6.5 μm × 6.5 μm), high speed (up to 3,000 fps) sCMOS camera for super resolution acquisition, and a CCD camera (1,392 × 1,040 pixels) for widefield imaging. Excitation lasers include 1 W 640 nm (excitation wavelength for Alexa Fluor 647), 1 W 561 nm (for CF 568) and 1 W 488 nm (for Alexa Fluor 488), as well as a 100 mW 405 nm activation laser, enabling excitation densities of approx. 5 kW/cm^2^; with epi-illumination at the sample site. All images were acquired using a 60× 1.2 NA water-immersion Olympus objective. Prior to imaging, an experimental point-spread function (PSF) was generated using 200 nm Tetraspeck microsphere beads (Thermo Fisher Scientific, Waltham, MA, USA) to create a response function of the microscope (experimental point spread function, PSF) to fit single molecule signals and align the individual biplane and color channels.

The bead-sample was prepared on a high-resolution coverslip of the thickness #1.5H (diameter 25 mm, Electron Microscopy Science, Hatfield, PA, USA) by coating the center of the glass with poly-L-lysine (Sigma–Aldrich St. Louis, MO, USA, 10 min incubation). Once dried, 30 μl of a well-sonicated (>10 min) 1:20 Tetraspeck dilution in ddH_2_O was added to the center and aspirated with a pipette after 10 min. The sample was then immersed in a drop of Immersol W 2010 (Carl Zeiss, Jena, Germany) and sealed with a coverslip and nail-polish.

An experimental PSF from orange and red channels was acquired using the calibration module in the Vutara SRX software (6.04.02), allowing a lateral registration of the biplane focus planes and the different color channels to each other with a root mean squared error (RMSE) of <5 nm.

### *d*STORM Imaging and Localization Process

Brain sections were transferred from the Scale U2 buffer to 0.1 M TBS and rinsed on an orbital shaker for 5 min before being placed flat atop a #1.5H circular cover glass (diameter 25 mm), with a small paint brush and left to dry completely. Imaging was performed in buffer containing reducing agent to enable reversible photoswitching of the utilized fluorescent dyes. The buffer consists of 20 mM *β*-Mercaptoethylamine (MEA) in 50 mM Tris-HCl (pH 8) + 10 mM NaCl, 1% (v/v) *β-mercaptoethanol* (BME; Sigma–Aldrich, St. Louis, MO, USA) and a 1× Glucose-oxidase (Gloxy) solution, all diluted in a buffer containing 50 mM Tris-HCl (pH 8), 10 mM NaCl and 10% (w/v) glucose. The Gloxy solution was prepared as a 50× stock containing 8440 AU of glucose oxidase type VII from Aspergilius (Sigma–Aldrich, St. Louis, MO, USA) and 70200 AU of catalase from bovine liver (Sigma–Aldrich) in a buffer containing 50 mM Tris-HCl (pH 8) and 10 mM NaCl. A drop of the imaging buffer was added onto the section and the tissue was left to re-hydrate and expand for 10 min to prevent movement during imaging. A second #1.5H cover glass was added on top of the section for immobilization and sealed to prevent further oxygen influx that facilitates photo damage and therefore bleaching of the fluorophores. The tissue construct was then placed into an AttoFlour imaging chamber (Thermo Fischer) and fixed on the sample holder to minimize drift. The switching buffer was replaced every 1.5 h to ensure a stable pH and therefore reproducible localization rates.

**Figure 2:**
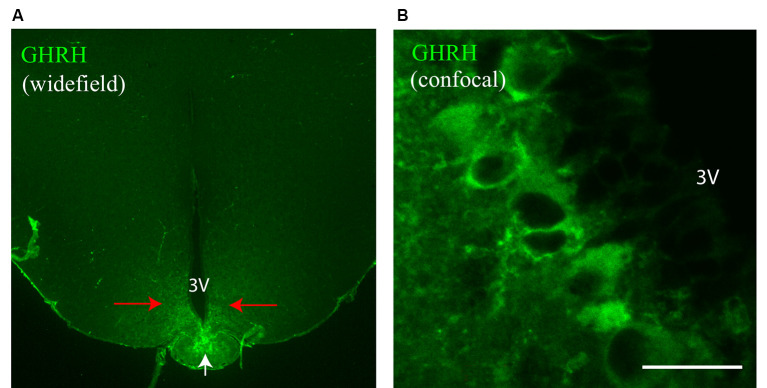
GH-Releasing Hormone (GHRH) neurons in the mouse brain. **(A)** A widefield overview image of a mouse brain section stained with an antibody against GHRH. The staining is specific to the arcuate nucleus (red arrows) and the median eminence (white arrow). **(B)** A confocal image of GHRH-stained neuronal bodies in the arcuate nucleus. 3V, third ventricle. Scale bar = 20 μm.

In the arcuate nucleus of the hypothalamus, the region of interest (ROI) was identified *via* fluorescence of GHRH-positive neurons and its lateral location with regards to the third ventricle (Figure 2A). The ROI was then randomly scanned on both sides of the third ventricle and each field exhibiting a triple labeling of GHRH with its pre-and post-synaptic markers (on average, between 13 and 20 fields (neurons) on each side of the third ventricle for a total of 26–40 neurons per section) was chosen for further super-resolution imaging, such that each super-resolved image contained synapses contacting the soma of one GHRH neuron. On average, 3–5 brain sections were imaged per animal. Widefield reference images were captured for the triple-labeled GHRH neurons before super-resolution imaging. Pre- and post-synaptic markers were then imaged sequentially with a 561 nm laser at 7.5 kW/cm^2^ and a 640 nm laser at 6 kW/cm^2^, both at 20 ms exposure time for 10,000 frames each. Single Molecule Localization (SML) was performed using the Vutara SRX software, allowing 3D localization by fitting biplane-signals to an experimental PSF. The fit window was set to 99.8 nm × 99.8 nm, and we allowed signal accumulation in consecutive frames if the localization center was shifted less than 4 pixels. If the same fluorophore was “on” more than 10 consecutive frames, it was considered a fiducial marker and used for drift correction. No maximum limit was set for the number of particles to be localized in a given frame.

### DBSCAN Cluster Analysis

**Figure 3:**
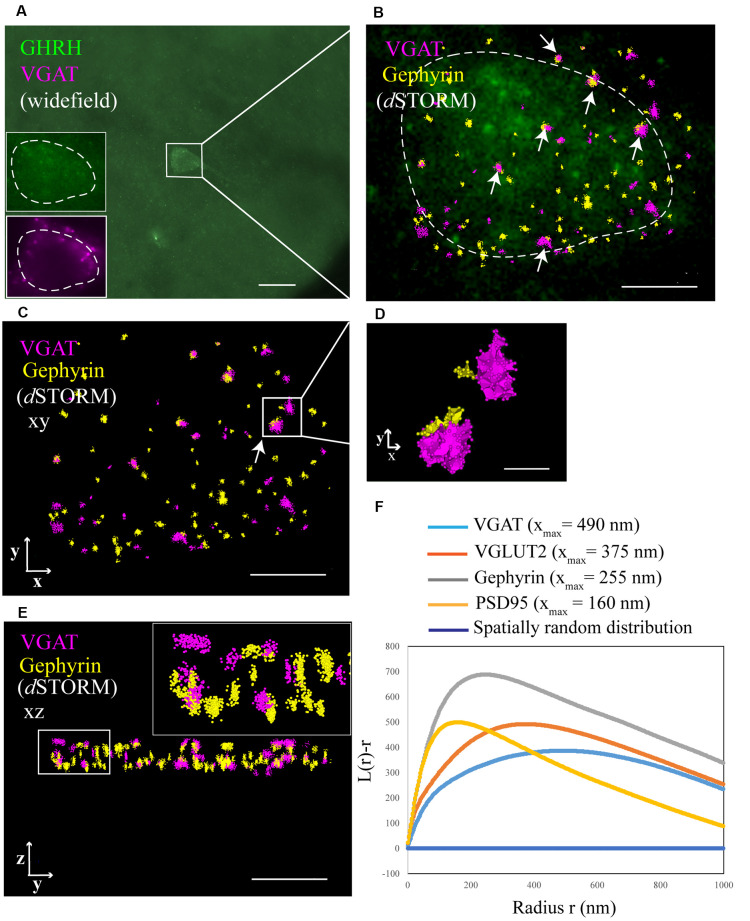
Identification of synaptic inputs to GHRH neurons with *d*STORM. **(A)** An overview widefield image of a GHRH-positive neuron (AF488, green) in the arcuate nucleus of the hypothalamus and two widefield zoomed-in images of the same GHRH cell (left bottom corner, top insert) and of a pre-synaptic marker VGAT (AF647, magenta; bottom insert). **(B)** Enlarged view of the reference GHRH cell shown in panel **(A)**, with inhibitory VGAT and Gephyrin (post-synaptic) clusters imaged using *d*STORM and rendered with a Density-Based Spatial Clustering of Applications with Noise (DBSCAN) algorithm, displayed as pointcloud. Synaptic inputs to GHRH neurons are shown with arrows and identified as opposing pre- and post-synaptic clusters with an inter-cluster distance of <250 nm. **(C)** A pointcloud visualization of synaptic markers from panel **(B)** without the reference GHRH cell in the background, shown in the XY-plane. **(D)** Enlarged clusters of VGAT and Gephyrin from panel **(C)** to highlight lateral resolution. **(E)** YZ-plane depiction of VGAT and Gephyrin. The axial resolution allows 3D distance analysis. Scale bars: **(A)** = 20 μm **(B,C,E)** = 4 μm, **(D)** = 500 nm. **(F)** A general cluster test by Ripley’s H analysis, represented as a normalized Ripley’s H function (L(r)-r) for indicated synaptic proteins. The peak indicates the highest degree of aggregation at specific interacting distance r compared to a spatially random distribution with L(r)-r of 0 (dark blue line). Pre-synaptic proteins VGAT and VGLUT2 cluster at higher radii (490 nm and 375 nm, respectively), as compared to post-synaptic proteins Gephyrin and PSD95 (255 nm and 160 nm, respectively).

**Figure 4:**
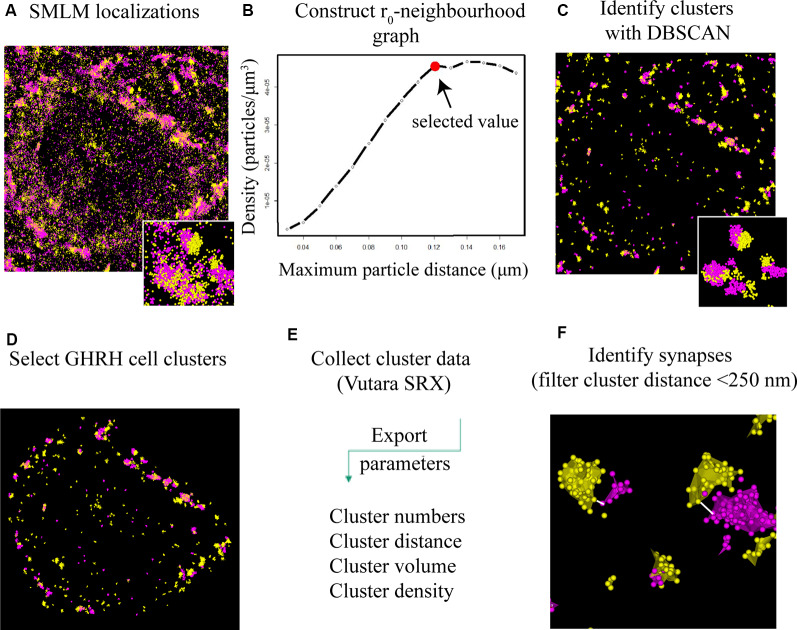
DBSCAN extraction of synapses from SMLM localization data. **(A)** All single molecule localization microscopy (SMLM) localizations of VGLUT2 (magenta) and PSD95 (yellow), displayed as a pointcloud visualization. The data contains clusters (synaptic and non-synaptic) and single localizations (non-clustered proteins, unbound antibodies). **(B)** Serial parameter scan for DBSCAN: the mean value of the localization density per identified cluster is plotted against the increasing DBSCAN distances (r_0_) while the minimum particle count is kept constant to 6. The plateauing curve indicates stable clustering parameter. We choose the beginning of the plateau as the ideal r_0_ value for the specific protein population. **(C)** DBSCAN algorithm filters data based on the input parameters and displays resulting clusters. **(D)** Synaptic clusters associated with a GHRH cell are selected manually (based on an overlay with a GHRH reference image, as shown in Figure 3B). **(E)** Various quantitative cluster data of interest is exported to an Excel file. **(F)** An in-house-built algorithm for the software “R” filters full synapses based on their inter-cluster distance between pre-and post-synaptic clusters of <250 nm.

To identify and extract inhibitory and excitatory synaptic clusters from all recorded localizations, a Density-Based Spatial Clustering of Applications with Noise (DBSCAN) algorithm, proposed by Ester et al. (1996) was utilized and run through the Vutara SRX software. The DBSCAN algorithm assigns localizations to a cluster based on a threshold for the minimum particle number of neighboring localizations found within a maximum particle distance from the current localization. All unassigned localizations are excluded. The DBSCAN variables are determined by keeping the minimum number of neighbors constant at a value of 6 as recommended for three dimensional data in (Ester et al., 1996) and varying the distance radius. Therefore, the density of particles per μm^3^ (a parameter of the determined cluster) was plotted against a series of increasing distance values (DBSCAN variable; Figure 4B). The advent of a plateau in the distribution indicates that a cluster population is detected and can be used to indicate a reproducible value for the distance variable of DBSCAN. Cluster parameters such as area/volume and density were computed using a non-convex alpha shape hull. The alpha shape radius was set at 0.1 μm. Next, clusters that contacted GHRH neurons were selected by overlaying the widefield GHRH reference image with the super-resolved localizations that are assigned to a cluster. Since the GHRH staining delineates the soma of neurons, cell borders were established around the hormone staining, as shown in Figure 3B. Synapses were identified by a co-occurrence of a pre- and post-synaptic marker clusters that are less than 250 nm apart. The distance is defined as the space in between the closest particle of each cluster identified with an algorithm that enables relative distribution assessment of STORM localizations called STORM-RLA (Veeraraghavan and Gourdie, 2016). The cluster distance filtering was executed *via* an in-house-built “R”-code. The distance was chosen by estimations of synaptic protein distances as described in Dani et al. (2010) and because protein markers used in our study included vesicular transporters (VGAT and VGLUT2) and their axial positions were not earlier reported in Dani et al. (2010), the known size of a synaptic vesicle (~40 nm) was considered and added to the threshold for a final value of 250 nm. All data was exported into an Excel file and statistical analysis was performed using two-way ANOVA combined with a Bonferroni post-test.

## Results

### Pulsatile GH Secretion in Mice

With this project, our goal was to quantify the numbers—and study more novel aspects—of excitatory and inhibitory synapses that contact growth hormone-releasing-hormone (GHRH) neurons during peak and trough levels of circulating growth hormone (GH) in mice. A schematic representing tissue extraction and GH level measurement is depicted in Figure 1A. To validate the ELISA GH measurement assay, we obtained 6-h profiles based on 20-min interval sampling of circulating GH levels of three adult male mice (Figure 1B). A regular periodicity of pulsatile GH secretion was observed with profiles revealing a 2-h multicomponent peak based on peak-doublets (mouse 1) or shoulders (mouse 2 and 3) and there was a strong concordance in secretion timing (between ~11:20 and ~13:20), followed by a low baseline secretory period of similar duration. These data are comparable to observations by Steyn et al. (2011) in mice and similar to measures obtained earlier in rats (Tannenbaum and Martin, 1976). We then sampled more mice at 15–20-min intervals with at least five GH measurements before mouse perfusion and extracted a total of six brains, three corresponding to trough- and three to peak- levels of circulating GH (Figures 1C,D, respectively). Circulating levels of GH, obtained 15 min prior to sacrifice and tissue extraction are represented by the last time point on each individual graph in Figures 1C,D.

### GHRH Neurons in the Mouse Brain

Earlier studies utilizing *in situ* hybridization of GHRH gene expression in the mouse brain by Suhr et al. (1989) revealed that the hormone’s mRNA is restricted primarily to the arcuate nucleus of the hypothalamus. Furthermore, in an eGFP-GHRH transgenic mouse, it was shown that the peptide is transported from cell bodies in the arcuate nucleus to varicose fiber terminals in the median eminence, where the hormone is released (Balthasar et al., 2003). Therefore, to specifically label GHRH neurons in the mouse brain, we first collected tissue that contained the mediobasal hypothalamus (MBH) with the arcuate nucleus, as described in Materials and Methods. We then used an antibody against GHRH and observed specific staining of GHRH neurons in the arcuate nucleus (Figure 2A, red arrows; Figure 2B) and GHRH neuronal fiber terminals in the median eminence (Figure 2A, white arrow). No other regions of the MBH were labeled with the anti-GHRH antibody. Negative controls for all fluorescent markers used in the study were performed (GHRH, VGAT, Gephyrin, VGLUT2, PSD95 and their corresponding secondary antibodies) and revealed the absence of unspecific binding and low background fluorescence levels (Supplementary Figures S1A,B).

### *d*STORM and DBSCAN Allow for Quantitative Imaging and Analysis of Synapses That Contact GHRH Neurons

To better understand the mechanism of GH pulsatile release, we looked at the synaptic connectivity of GHRH neurons in the arcuate nucleus of the hypothalamus during peaks and troughs of GH secretion in the mouse. Figure 3A represents a sample widefield image of a GHRH neuron and an inhibitory pre-synaptic marker VGAT. However, as seen in the two inserts in Figure 3A, due to the limited resolution of conventional fluorescence microscopy, it would be difficult to quantitatively assess synapse numbers and other cluster parameters. Therefore, we utilized *d*STORM imaging of pre-and post- synaptic inhibitory and excitatory markers of GHRH neurons combined with a DBSCAN algorithm to quantify the numbers of inhibitory and excitatory synapses, as shown in Figures 3B,C. The improvement in resolution can be clearly seen in Figure 3D. To ensure a more accurate assessment of synaptic cluster numbers, we utilized the Vutara 350 (Bruker) equipped with a biplane module allowing 3D super-resolution imaging of 1 μm sectioning, as shown in Figure 3E with synapses revealed at different depths. To describe the degree of clustering for the proteins of interest, we utilized the normalized Ripley’s H function (Figure 3F). This is a descriptive statistic approach that allows to investigate the spatial homogeneity of points in a data set. Clustering is identified if the average number of points within a distance *r* of another point is statistically greater than what is expected for a random distribution (Kiskowski et al., 2009). Distribution curves of the post-synaptic density markers PSD-95 and Gephyrin follow a similar trend, showing a peak that indicates high-order clustering at small radii (160 nm for PSD 95 and 255 nm for Gephyrin). The distribution of pre-synaptic vesicular protein markers VGLUT2 and VGAT indicate bigger cluster with a maximum of aggregation at higher radii (375 nm for VGLUT2 and 490 nm for VGAT). This trend is possibly due to the size of the synaptic vesicle (40 nm) and the clustering at active zones. The difference in cluster size between pre- and post-synaptic proteins can be also seen in Figure 3D, with pre-synaptic clusters bigger than post-synaptic ones. The degree of clustering between animals and between the peak and trough concentration values of GH was comparable (data not shown). *d*STORM data acquisition relies on repetitive detection of a target bound fluorophore resulting in a localization nanocluster representing one fluorophore. This can aggravate the distinction of expected signal of localizations from unspecifically bound fluorophores (Figure 4A shows all recorded localizations surrounding the soma of a GHRH cell). Therefore, it is important to find suitable parameters for the cluster algorithm that represents the structure of interest in a reproducible manner. Here, we utilized DBSCAN, as shown in Figure 4. For the selection of parameters, the r_0_ neighborhood graph (Figure 4B) was used to select a value for the maximum particle distance to form a cluster. These values varied between animals, ranging from 0.10 μm to 0.16 μm. The minimum number of particles to form a cluster was set to 6. DBSCAN analysis of the synaptic localization data facilitated a clear extraction of clusters (Figure 4C). In comparison, we can observe the same structure boxed in the unfiltered *d*STORM data visualized in Figure 4A. Removal of clusters that did not contact GHRH-positive neurons (by deleting clusters outside of the cluster overlay zone with a GHRH widefield image) allowed selection of only clusters of interest (Figure 4D). Various cluster data was collected in the SRX software (Figure 4E) and a quantitative assessment of the inter-cluster distance was used to select synapses based on a threshold of 250 nm (Figure 4F). As such, the data presented here underscores the potential of *d*STORM to quantitatively assess the synaptic architecture of a cell of interest in the neuroendocrine circuit.

### Quantitative Assessment of Synapse Numbers and Other Synaptic Parameters

**Figure 5:**
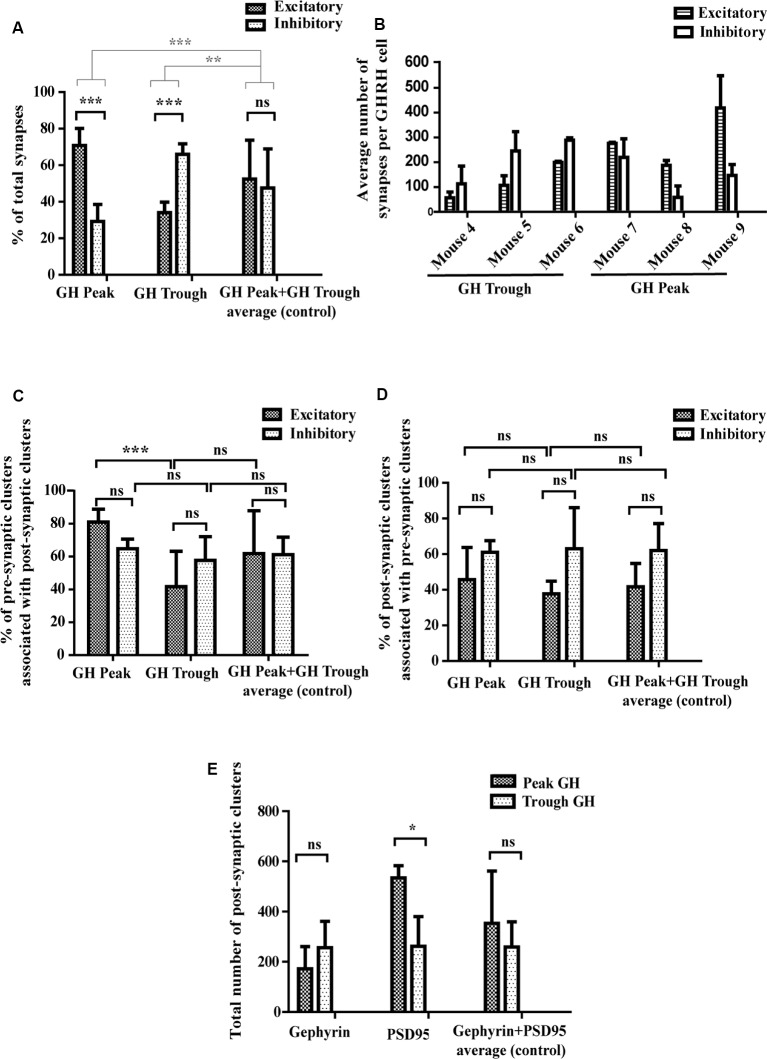
Quantitative assessment of synaptic parameters. **(A)** Synapse numbers during peaks and troughs of GH secretion. During high hormone levels (“GH Peak”), 69% of all synapses are excitatory and 31% are inhibitory. During low hormone levels (“GH Trough”), 34% of all synapses are excitatory and 66% inhibitory. There is no significant difference in the percent of excitatory and inhibitory synapses in the control condition (“GH Peak+GH Trough”). Black significance bars represent the change in synapse numbers (as % of total synapses) between the three conditions. Gray significance bars represent the significance levels of the percent change of excitatory and inhibitory synapse numbers at GH Peak and GH Trough as compared to the percent change of the average (control). Black significance bars: ANOVA-2 *p*_(interaction)_ < 0.001; after Bonferroni *post hoc* test correction *p*_(GH Peak)_ < 0.001, *p*_(GH Trough)_ < 0.001. Gray significance bars: ANOVA-1 *p* < 0.001; after Tukey *post hoc* test *p*_(percent change GH Peak vs. percent change Average)_ < 0.001, *p*_(percent change GH Trough vs. percent change Average)_ = 0.005. **(B)** Absolute synapse numbers in each mouse per GHRH cell. Although numbers of excitatory and inhibitory synapses varied between mice [excitatory synapse numbers ranged from 45 (in mouse 4) to 372 (in mouse 9) and inhibitory synapse numbers ranged from 47 (in mouse 8) to 255 (in mouse 6)], the ratios of excitatory to inhibitory synapses between each mouse were more conserved. Data displayed as means ± SD. **(C)** Synapse-forming pre-synaptic clusters. During GH Peak, 81% of excitatory pre-synaptic clusters are associated with excitatory post-synaptic clusters, forming a synapse (the remaining 19% are “free” pre-synaptic clusters). This changes during GH Trough, where only 41% of pre-synaptic clusters are associated with post-synaptic clusters (the remaining 59% are “free” pre-synaptic clusters). This is not seen in inhibitory clusters, where levels remain similar (65% of pre-synaptic clusters associate with post-synaptic clusters during peaks and 58% during troughs). No change is observed in the control data. ANOVA-2 *p*_(GH Peak vs. GH Trough)_ < 0.001; after Bonferroni *post hoc* test correction *p*_(GH Peak Excitatory vs. GH Trough Excitatory)_ < 0.001. **(D)** Synapse-forming post-synaptic clusters. No significant difference was found between the percent of post-synaptic clusters that associate with pre-synaptic clusters to form synapses during GH Peak and GH Trough. ANOVA-2 *p*_(Excitatory vs. Inhibitory)_ = 0.008; after Bonferroni *post hoc* test correction *p*_(all factors)_ > 0.62. However, the absolute number of post-synaptic excitatory PSD95 clusters increased during GH Peak levels from an average of 262–534 clusters, as presented in **(E)** with ANOVA-2 *p*_(GH Peak vs. GH Trough)_ = 0.03; after Bonferroni *post hoc* test correction *p*_(PSD95 GH Peak vs. PSD95 GH Trough)_ = 0.02. No changes were seen in the control data, in both **(D,E)**. All data is displayed as means ± SD using two-way ANOVA with significance values at 0.12 (ns), 0.033 (*), 0.002 (**), <0.001 (***).

We have reported earlier that synaptic connectivity varies in parallel with the ultradian rhythm of GH secretion in rats, using an electron microscopic approach (Stroh et al., 2009), where synapses were counted visually in one plane. Here, we confirm and extend our findings to mice, revealing increased excitatory input to GHRH neurons during peak levels of circulating GH, while increased inhibitory inputs are detected when GH levels are low. Specifically, we found that when blood hormone levels were high, 69% of all synapses that contact GHRH neurons were excitatory and 31% inhibitory (*p* < 0.001; *n* = 3 animals; Figure 5A, “GH Peak”). However, when blood hormone levels dropped we observed a rapid GHRH circuit rewiring, where now 34% synapses were excitatory and 66% inhibitory (*p* < 0.001; *n* = 3 animals; Figure 5A, “GH Trough”). No change was observed in the control group, where we combined all available data of excitatory and inhibitory synapses for GH Peak and Trough to scramble peak and trough-associated differences [Figure 5A, “GH Peak+GH Trough (…)”]. When comparing the absolute inhibitory and excitatory synapse numbers in each mouse (Figure 5B), we noted a significant variation between animals (thus, the data from all mice could not be combined for statistical reasons). However, the ratios of excitatory and inhibitory synapses between animals were mainly conserved. Moreover, the increase in excitatory synapse ratio during peak GH, as seen in Figure 5A, seems to be the result of an increase in the absolute numbers of excitatory synapses as well as the decrease in the absolute numbers of inhibitory synapses, as per visual assessment of the data from all graphs in Figure 5B (all bars for “Excitatory” synapses are higher at GH peak and all bars for “Inhibitory” synapses are lower during GH trough). It is known that synaptic vesicles assemble from smaller, reserve clusters situated more distal to the release site, into bigger release clusters at the release site (Pechstein and Shupliakov, 2010; Vaden et al., 2019). Thus, we hypothesized that the fast circuit rewiring observed between the peak and trough of GH release in the arcuate nucleus might be a result of mobilization and clustering of the reserve-pool of pre-synaptic vesicles into the pre-synaptic membrane. To this end, we counted the numbers of fully evolved synapses (from here-on dubbed “full synapse”). These are defined as an apposition of a pre- and post-synaptic cluster with an inter-cluster distance of <250 nm (as described earlier). The amount of full synapses has been compared to the numbers of all presynaptic clusters including free pre-synaptic clusters not associated with a synapse and those forming full synapses. All results are calculated following the equation:

$$\%\text{of pre-synaptic clusters associated with post-synaptic clusters =}\frac{\text{number of full synapses}}{\text{number of all pre-synaptic clusters}}*100\%$$

We found that during GH peak secretion, 81% of pre-synaptic excitatory VGLUT2 clusters had a post-synaptic excitatory “partner” labeled with an anti-PSD95 antibody, thus forming a full synapse (Figure 5C, “GH Peak,” Excitatory). However, during GH trough secretion, only 41% of all pre-synaptic VGLUT2 clusters had a PSD95 post-synaptic partner (Figure 5C, “GH Trough,” Excitatory; *p* < 0.001). No differences were seen in the control group [Figure 5C, “GH Peak+GH Trough (…)”]. We found no difference in the association of pre-synaptic clusters with post-synaptic ones in the inhibitory system, where 65% and 58% of all pre-synaptic VGAT clusters formed a full synapse during high and low GH levels, respectively (Figure 5C, “GH Peak,” Inhibitory, vs. Figure 5C, “GH Trough,” Inhibitory). We repeated the analysis for post-synaptic clusters that associate with pre-synaptic clusters (as opposed to pre-synaptic clusters associating with post-synaptic ones, see above) and found no significant differences in both, excitatory and inhibitory synapses, between GH trough and peak levels (Figure 5D). This was puzzling, since we expected that the observed increase of pre-synaptic clusters that form synapses between trough and peak of GH secretion would require a parallel increase in the association of post-synaptic clusters with pre-synaptic ones to account for the changing synapse numbers. As such, we reasoned that the absolute number of post-synaptic excitatory clusters increases, such that post-synaptic clusters associating with pre-synaptic ones to form new synapses are balanced out by new post-synaptic clusters (thus maintaining a constant cluster ratio). Since cluster numbers were comparable between animals (data not shown), they could be combined for a direct comparison. Indeed, we found 262 PSD95 clusters present during trough- and 534 PSD95 clusters during peak of GH secretion (Figure 5E, “PSD95,” Trough GH vs. Peak GH; *n* = 3 animals; *p* = 0.02). There was no significant difference in the total cluster number for Gephyrin between trough and peak of GH (Figure 5E, “Gephyrin,” Trough GH vs. Peak GH), which supports our earlier findings of no changes in the percentage of pre-synaptic clusters that associate with post-synaptic ones in the inhibitory system.

## Discussion

For decades, structural synaptic studies have relied on electron microscopy, a technique providing the resolution to visualize individual synapses. In the neuroendocrine circuit, such studies were mainly done using immunogold labeling of a target cell followed by a visual analysis/quantification of associated synapses in one plane of view, with limitations in multi-component 3D analysis. As a result of extensive sample processing, the image quality suffered and a small field of view required many rounds of imaging. Fluorescence microscopy overcomes these limitations by allowing high-throughput imaging with high target specificity, but its diffraction-limited resolution does not allow for the quantitative analysis of synaptic components (Maglione and Sigrist, 2013). Therefore, protein localization at a nanometer-level is challenging. However, the advent of super-resolution fluorescence microscopy by SMLM enabled target-specific visualization of proteins at the nanometer scale resulting in a multitude of biologically-relevant discoveries (Nair et al., 2013; Andreska et al., 2014; Ehmann et al., 2014; Rahbek-Clemmensen et al., 2017; Lorenzo et al., 2019; Reinhard et al., 2019; Siddig et al., 2020). Here, we present a first-to-date super-resolution imaging of GHRH neuroendocrine cells in organotypic tissue to quantitatively assess the synaptic architecture in the GH-regulating circuit by utilizing *d*STORM paired with DBSCAN (Density-Based Spatial Clustering of Applications with Noise) cluster analysis. We employ mice with a known GH secretion status pre-measured with sandwich ELISA prior to fixation. Combining these techniques enabled us to quantitatively demonstrate that GHRH neurons in the arcuate nucleus of rodent hypothalamus receive increased excitatory input during peak levels of GH secretion. This result confirms our earlier findings by TEM measurements (Stroh et al., 2009). Additionally, we find excitatory synapse numbers dropping during periods with low levels of circulating GH, indicating rapid circuit rewiring within a single period of the GH secretion cycle. Lastly, we show that more pre-synaptic excitatory clusters associate with post-synaptic excitatory clusters forming a full excitatory synapse and that the absolute number of post-synaptic excitatory clusters increases during peak levels of GH secretion, while no increase of synapse formation is detected in the inhibitory system. We therefore propose that a mobilization of free excitatory pre-synaptic “reserve-pool” clusters towards the pre-synaptic membrane unfolds during peak hormone secretion periods. This appears to happen in conjunction with the *de novo* emergence of additional post-synaptic excitatory synaptic densities to form excitatory synapses. Ultimately, this might be a mechanism contributing to the regulation of pulsatile ultradian GH secretion.

Interestingly, GHRH neurons do not display rhythmicity at the electrical level: patch-clamp studies *in situ* did not reveal the presence of electrical oscillations in cell bodies (Baccam et al., 2007). Furthermore, factors such as hypoglycemic challenge or stimulation of the GH axis (central or peripheral) increase GHRH neuron spike discharge (Stanley et al., 2013; Osterstock et al., 2016). As such, the up- or down-regulation of the firing activity of GHRH neurons appears to play an important role in the control of pituitary GH secretion. It has been shown that agonists of somatostatin receptor irregularly suppressed GHRH neuron electrical activity leading to slow oscillations within a population, as soon as 12 min after octreotide superfusion in male mice (Osterstock et al., 2016). This happens *via* initial hyperpolarization through activation of K+ channels followed by a sst1/sst2-receptor dependent unbalancing of glutamatergic and GABAergic synaptic inputs (Osterstock et al., 2016).

At the structural level, the existence of rapid structural plasticity in neuroendocrine circuits has initially been shown in the oxytocin system by Theodosis et al. (1981), with rapid synapse formation and elimination in response to prolonged activation (parturition, lactation, chronic dehydration). The proposed mechanism underlying this rewiring is a glial ensheathment of neurons (or lack thereof) that prevents (or allows) synapse formation. More recently, in the arcuate nucleus of the tuberal hypothalamus, synaptic structural plasticity has been shown to take place in the circuitry controlling feeding, where changes in the number of excitatory and inhibitory synapses contacting the perikarya of NPY and POMC neurons were detected 6 h post leptin injection (Pinto et al., 2004). Combined, these observations suggest that activity-dependent circuit rewiring may drive or inhibit neuronal secretion in the neuroendocrine hypothalamus.

Here, we report rapid changes in synapse numbers contacting the perikarya of GHRH neurons when circulating GH levels rise from baseline. We only analyzed synapses on the soma of GHRH, since the hormone is highly concentrated there and only low levels are present as irregularly appearing “puncta” in axons of GHRH neurons (Balthasar et al., 2003), through which GHRH is rapidly transported to varicose fibers in the median eminence (a region with the highest concentration of GHRH). Moreover, in brain sections, axons have a “curved” trajectory and it is difficult to find a single GHRH neuron with all its projections in one plane of sectioning. Furthermore, no hormone is present in the dendrites of GHRH neurons (Balthasar et al., 2003), therefore it would be hard to asses which synapses in the vicinity of cell bodies actually contact GHRH cells (without introducing a fourth immunofluorescent label). By solely assessing somatic synapses, we ensured their association with the cell of interest. Furthermore, we did not expect to see opposing effects (or opposing synapse ratios) in axons to those in soma, as it would lead to an unnecessary energy expenditure in the cell.

Because we sacrificed and extracted brains around the expected rise of circulating GH levels between 11:00 and 12:30 (see reference profiles in Figure 1B and sacrification time points in Figure 1C and Figure 1D), we can conclude that the inversion of excitatory to inhibitory synapse ratios that is associated with the switch from baseline to rising GH levels, may typically take place in less than 90 min. This astoundingly rapid rewiring seems to be based on a combination of recruitment of “free” clusters of pre-synaptic components and possible *de novo* assembly of post-synaptic densities. The former mode of recruitment from pre-assembled units, at least pre-synaptically, is in line with other reports (reviewed in Garner et al., 2002). Notably, the formation of a pre-synaptic terminal has been previously shown to occur in only 10–20 min and the recruitment of post-synaptic receptors takes place 90 min after cell-cell contact (Garner et al., 2002). Interestingly, by comparing the numbers or pre-synaptic “free” clusters of VGLUT2 (a putative pre-assembled component of a future glutamatergic synapse) with “full” synapses (defined as an apposition of pre- and post-synaptic clusters with an inter-cluster distance of <250 nm, as described earlier) along the GH secretion time course, we noticed that free pre-synaptic clusters drop in number while full synapse frequencies rise when GH levels are high (GHRH neurons are stimulated). We did not observe such changes for the post-synaptic side. However, values are reported as ratios of all post- (or pre-) synaptic clusters to those clusters that form synapses and although this ratio remained unchanged between peak and trough of GH levels for the post-synaptic clusters, the absolute numbers of PSD95 clusters increased during peak levels of GH secretion [and were conserved between mice (data not shown)]. It is worth noting that while reporting the absolute numbers of synapses per GHRH cell in Figure 5B, we noted a significant variation in the *numbers* of excitatory (or inhibitory) synapses between mice (the *ratios* of excitatory to inhibitory synapses were more conserved and served as a basis for all analysis in the present study). This variation in synapse numbers between animals could be due to many factors, from those of experimental nature (such as the density of immunolabeling) to physiological factors, such as the brain size and the size of GHRH cells, or other particular differences in the amplitude of GHRH stimulation/inhibition of each mouse (the last possibly hinted by the difference in absolute values of the plasma GH concentrations between mice). Those of experimental nature would apply equally to both excitatory and inhibitory synapses in each animal, making synapse ratios a more suitable parameter for analysis; those of physiological nature were not a subject of this study and were not measured.

Looking at the increase of PSD95 cluster numbers, it is unclear how new cluster formation could have occurred, considering the fairly short time frame. However, it is known that PSD95 is directly involved in synaptic plasticity and interestingly, as proposed by Gray et al. (2006) using *in vivo* two-photon microscopy in the neocortex, synaptic PSD95 turns over rapidly (22–63 min) and exchanges with PSD95 in neighboring spines by diffusion. Here, we solely report a rapid increase in PSD95 cluster numbers between trough and peak of GH levels. The mechanism responsible for the observed changes remains unclear and would be an interesting topic for future investigations.

## Data Availability Statement

The datasets generated for this study are available on request to the corresponding author.

## Ethics Statement

The animal study was carried out according to the guidelines of the Canadian Council on Animal Care. It was reviewed and approved by the Animal Care Committee of McGill University.

## Author Contributions

KB, WA and TS designed all experiments, which were performed by KB. KB generated the figures. WA and TS assisted in mouse perfusions and tissue extraction. SA assisted in setting up *d*STORM imaging and analysis. TD programmed a code in the “R” statistical software. PM trained KB in mouse blood collections and ELISA procedures. K-FS provided mice for the study and assisted in interpreting results. KB and TS wrote the manuscript. WA, SA and K-FS provided input and edited the initial draft of the manuscript. KB implemented reviewer comments. SA and TS edited the final draft of the manuscript.

## Conflict of Interest

The authors declare that the research was conducted in the absence of any commercial or financial relationships that could be construed as a potential conflict of interest.

## Supplementary Material

FIGURE S1 Negative controls for immunofluorescence stainings. **(A)** Control immunofluorescence labeling for inhibitory synapses. Each column represents a confocal image of the arcuate nucleus with an antibody staining against a specific target (GHRH neuron, pre or post-synaptic markers). Rows represent an image of an immunofluorescent staining of either all three targets (triple “+”) or two targets (double “+,” single “−”) with all three secondary antibodies present. Clear signal is observed for the three-target immunofluorescence image and no unspecific secondary antibody binding is seen for all two-target controls. Scale bar = 50 μm. **(B)** Similar to **(A)** but for excitatory synapses.
