# Supplementary figures and images for: Ultradian Secretion of Growth Hormone in Mice: Linking Physiology With Changes in Synapse Parameters Using Super-Resolution Microscopy
Source: Front Neural Circuits. 2020 May 25;14:21. doi: 10.3389/fncir.2020.00021 (PMC7261915; doi:10.3389/fncir.2020.00021)

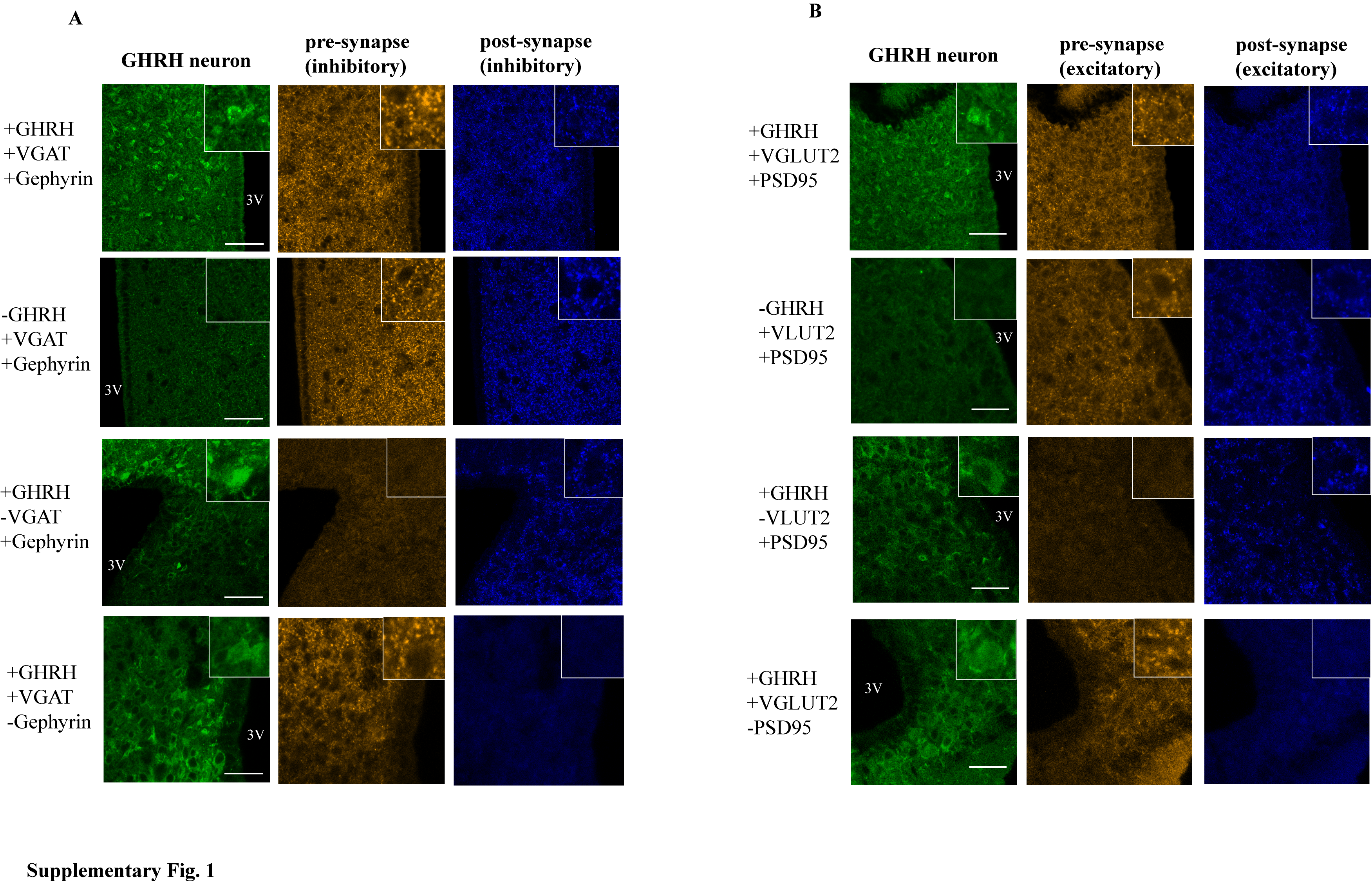

Supplement: FIGURE S1 — Negative controls for immunofluorescence stainings. (A) Control immunofluorescence labeling for inhibitory synapses. Each column represents a confocal image of the arcuate nucleus with an antibody staining against a specific target (GHRH neuron, pre or post-synaptic markers). Rows represent an image of an immunofluorescent staining of either all three targets (triple “+”) or two targets (double “+,” single “−”) with all three secondary antibodies present. Clear signal is observed for the three-target immunofluorescence image and no unspecific secondary antibody binding is seen for all two-target controls. Scale bar = 50 μm. (B) Similar to (A) but for excitatory synapses. [file Image_1.tif]
